# Turbidity reduction efficacies of seed kernels of Mango (*Mangifera indica*) genotypes in Uganda

**DOI:** 10.1016/j.heliyon.2023.e21415

**Published:** 2023-10-21

**Authors:** Charles Onyutha, Nancy Auma

**Affiliations:** Department of Civil and Environmental Engineering, Kyambogo University, P.O. Box 1, Kyambogo, Kampala, Uganda

**Keywords:** Water treatment, Turbidity removal, Natural coagulants, Mango genotypes

## Abstract

Alum and ferric salts as traditional chemical coagulants for turbidity removal in water and wastewater are expensive, and have known harmful effects. Thus, attempts to replace the chemical coagulants with safe and effective natural solutions are increasingly being made in terms of research studies to investigate the coagulation efficacies of various plants one of which is Mango (*Mangifera indica*). It is worth noting that *M. indica* has various genotypes of different origins across the world. In this study, eight (8) common *M. indica* genotypes in Uganda were identified, protein contents of their seed kernels determined, and coagulating efficacies investigated. Coagulation solution of each selected genotype was obtained by dissolving 5 g powder of the selected Mango seed kernel sample in 100 mL of distilled water. Next, 5 mL of this coagulant (or stock) solution was used to treat 200 mL of turbid water with turbidity ranging from 15 to 120 NTU. Using 0.01 M hydrochloric acid as an extraction solvent, protein contents of selected genotypes including Apple mango, Kate, Kent, Bire, Doodo red, Takataka, Kagoogwa, and Tommy Atkins were 38.02 %, 30.66 %, 15.94 %, 22.11 %, 21.50 %, 16.98 %, 16.36 %, and 17.87 %, respectively. Efficacies of coagulant from Apple mango, Kate, Kent, Bire, Doodo red, Takataka, Kagoogwa, and Tommy Atkins seed kernel samples were 92.2, 89.3, 66.0, 78.7, 76.9, 71.1, 68.9, and 73.1 %, respectively. Apple mango was the best performing genotype as a coagulant and this was followed by Kate. Coagulation efficacy was generally found to increase with increasing turbidity and/or coagulant's concentration. For instance, Apple Mango coagulant removed 16.7 %, 50.3 %, and 92.2 % of initial turbidity 15, 65 and 120 NTU, respectively. Kent removed 57.5, 66, and 69 % of initial turbidity 120 NTU using 5, 20, and 30 mL of stock solution, respectively. This study demonstrated the influence of the choice of a plant genotype on coagulation efficacy.

## Graphical abstract

**Figure:**
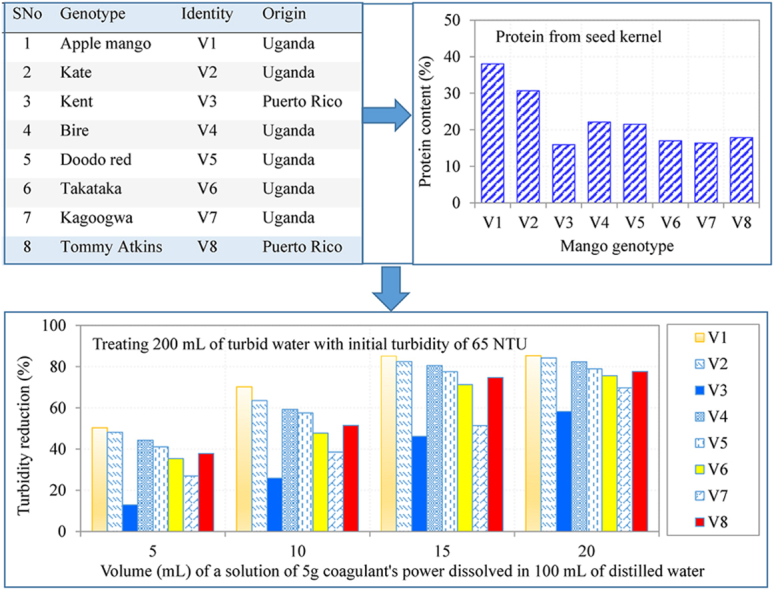

## 1 Introduction

Human health and drinking water quality are inextricably linked [1]. A sufficient (appropriate, safe, and accessible) supply of water is required for human survival. Surface water is the main supply of drinking water in developing countries. Water from such an exposed source is untrustworthy since it tends to contain lots of suspended particles thereby reducing its transparency, a phenomenon termed as turbidity. In many water sources, dissolved and suspended particles make up the majority of the pollutants. The majority of these suspended elements come from top soil erosion, mineral dissolution, and vegetation decomposition, as well as a variety of home and industrial waste outputs [2]. The suspended particles in terms of physical impurities can engender taste, colour, and smell to water thereby making it unacceptable for human consumption. The suspended particles can also likely serve as a home for pathogenic organisms. Thus, turbidity should be reduced to zero or nearly so for effective water disinfection.

Coagulation is one of the most important water treatment procedures for removing turbidity (or tiny suspended particles) from raw water. Because of their simplicity, alum and ferric salts have long been employed as traditional coagulants in water and wastewater treatment [3]. However, these chemical coagulants have a number of drawbacks that make their usage less attractive. Because of its limited or no degradability, harmful effects, and aluminium accumulation in the environment, sludge formed from water treatment utilizing aluminium salts, for example, causes disposal issues. These treatment chemicals tend to evidently remain in treated water yet they are potentially detrimental to human health [4]. Intestinal constipation, loss of memory, convulsion, abdominal colic, loss of energy, and learning difficulties may be caused by residual aluminium sulphate (alum) and polyaluminum chloride, whereas synthetic organic polymers, such as acrylamide, have neurotoxic and carcinogenic effects [5]. Determining the optimal alum dosage for a particular raw water source is especially challenging. Furthermore, alum is expensive in a developing and low-income countries.

Replacing chemical coagulants with effective and safe natural solutions is increasingly being considered a top priority in turbid water coagulation. Due to their safety, abundance, and cost effectiveness, several natural products derived from various plant parts including seeds, fruits, and leaves have exhibited properties which make them promising coagulants to be used as alternatives to chemical coagulants [6]. Some of the natural products include seeds and leaves of *M*. *indica* (Mango fruits), pods or seeds of *Okra (Abelmoschus esculentus),* seeds of *Moringa oleifera* [7], pods and seeds of *Tamarind* [8], leaves of *Acorn* [9]. Plants like Mangoes, African star apple, and African pear seeds contain active components such as tannins and natural polymers like proteins and starch that have been shown to cause coagulation [10]. Some plants also have ingredients such as saponins, flavonoids, coumarins, and phenols that have antibacterial properties against pathogenic organisms [10]. As a result, in addition to using these plants’ seeds for human health and livestock treatment, their extracts could be investigated further as possible water coagulant aids [10].

Efficiency of turbidity reduction of Duncan Mango was shown to vary with different turbidity levels [11]. Furthermore, the protein extraction efficiency of *M*. *indica* was found to be higher than many of other natural plants such as *Aesculus Hyppocastanum, Castanea sativa, Quercus robur, Quercus cerris, Quercus rubra* [11]. However, we posited in this study that the turbidity removal efficacy of *M*. *indica* could depend on the selected genotye. Thus, this study aimed at identifying the different genotypes of *M*. *indica* in Uganda, investigating their protein contents and turbidity reduction efficacies. The idea was to yield information for guiding the selection of the variety of *M*. *indica* that could be used as the most promising natural coagulant.

## 2 Materials and methods

### 2.1 Mango genotypes and seed powder preparation

#### 2.1.1 Selected *M*. *indica* genotypes

Information on the *M*. *indica* genotypes available in Uganda was formally obtained from the Department of Horticulture of the National Crops Resources Research Institute (NACRRI), Namulonge, Uganda. Mature fresh fruit samples of eight (8) *M*. *indica* genotypes (Table 1) available in the field of the Department of Horticulture at Ngetta Zonal Agricultural Research Institute, Lira, Uganda, were obtained for this study. Apart from their availability, these varieties were selected following guiding information regarding their yield performance in Uganda as assessed in a previous study [12].

**Table 1:** Mango genotypes used in this study.

SNo	Genotype	Identity in this study	Origin
1	Apple mango	V1	Uganda
2	Kate	V2	Uganda
3	Kent	V3	Puerto Rico
4	Bire	V4	Uganda
5	Doodo red	V5	Uganda
6	Takataka	V6	Uganda
7	Kagoogwa	V7	Uganda
8	Tommy Atkins	V8	Puerto Rico

#### 2.1.2 Preparation of *M*. *indica* seed powder

To prepare *M*. *indica* powder, mature fresh fruits were picked from Mango trees of each of genotypes listed in Table 1. Each fruit was washed with tap water and manually sliced through the exocarp and mesocarp to obtain the endorcarp (or seed). The seed was washed using tap water and further dried under the sun for a period of one month. During that month, the temperature of the location at which the seed was dried varied between 17 and 29 °C. The dry seeds were cut open to obtain the seed kernels which were further dried for about one month. The dried kernels were manually ground to fine powder using a mortar and pistol. The powder was sieved to obtain fine particles of appropriate size sieve of 300 μm aperture.

### 2.2 Determining the active coagulating component M. indica

#### 2.2.1 Preparation of extraction solvents

Three (3) extraction solvents including Tris-Hydrochloride (Tris-HCl), Phosphate Buffer Saline (PBS), and 0.01 M Hydrochloric acid (HCl) were used to extract the protein content in the selected *M*. *indica* varieties.

To obtain the first extraction solvent, 50 mL of Tris-HCl and 300 mL of sodium chloride (NaCl) were required to be mixed at a pH of 8.5. To do so, the masses of Tris-HCl and NaCl were determined to be 2 g and 4 g, respectively and they were thoroughly mixed with 300 mL of distilled water to dissolve the crystals. Next, 20 mL of the resultant solution was measured and placed in three labelled falcon tubes. The pH of the solution in each tube was recorded in triplicate.

To obtain the second extraction solvent, two (2) tablets of PBS were dissolved in 200 mL of distilled water in a conical flask. Next, 20 mL of the resultant solution was measured and placed in three labelled falcon tubes. Again, the pH of the solution in each tube was recorded in triplicate.

To obtain the third extraction solvent, 0.1 mL of 0.01 M HCL was mixed with 300 mL of distilled water. Next, 20 mL of the resultant solution was measured and placed in three (3) labelled falcon tubes. Finally, the pH of the solution in each tube was measured in triplicate.

#### 2.2.2 Extraction of protein content

The various *M*. *indica* genotypes were first labelled for the ease of identification. Next, 5 g of every *M. indica* genotype sample was mixed with 20 mL of each extraction solvent. Next, 5 g of the sample was homogenized with 5 mL of the extraction solvents by grinding thoroughly with sterilized mortar and pistol and poured into a falcon tube. The remaining 15 mL of each extraction solvent was topped up in the falcon tube to make the 20 mL volume. The resulting mixture was vortexed using a vortex mixer for five (5) mins and clarified by centrifugation at 6000 rpm for ten (10) mins at +4 °C. The supernatant/clarified organic extract was collected into a clean 50 mL falcon tube. The pH of the supernatant organic extract of the various samples for the different extraction solvents were measured and recorded in triplicate.

To analyse the total protein content in the various genotypes, Bradford buffer solution was used. To prepare the Bradford reagent solution, 100 mg of Coomassie Brilliant Blue G250 was weighed and poured in a conical flask. Next, 50 mL of 95 % Ethanol was measured and also poured in to the same conical flask. To the same mixture, 100 mL of 85 % Phosphoric acid was measured and added. The mixture was shaken till the resultant solution formed a bright blue colour. The volume of the resultant solution was raised to one (1) litre with distilled water. The resultant solution was then filtered and kept at +4 °C. From the centrifuge mixture, 1 ml of the organic extract of every genotype sample was picked using a pipette and put into a 50 mL falcon tube. Next, 5 mL of the Bradford reagent was got and added on to the sample extract in the falcon tube. The absorbance readings of the various samples were taken using the Spectrophotometer at 595 nM and recorded in triplicate using the equation y+0.0148=0.3458x where y is the known absorbance reading and x denotes the unknown protein.

### 2.3 Turbid water coagulation using M. indica

#### 2.3.1 Preparation of synthetic turbid water

**Fig. 1:**
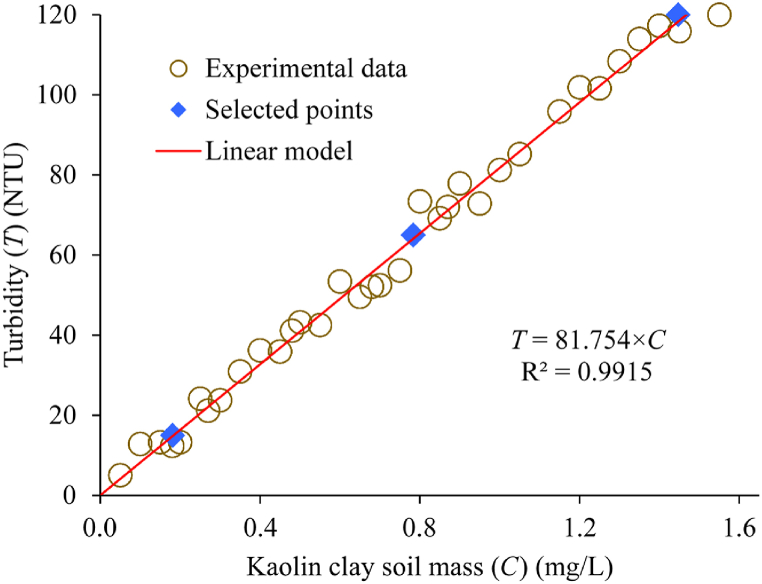
Kaolin clay soil mass *versus* resulting turbidity.

To create synthetic turbid water, a selected mass of kaolin clay soil between zero and 1.5 g was weighed and dissolved in a litre of distilled water. The solution was put in a jar test device and mechanically stirred rapidly and then gently at 300 and 40 rpm for five (5) and thirty (30) mins, respectively. The solution was left undisturbed for ten (10) mins and turbidity of the supernatant liquors determined in Nephelometric Turbidity Units (NTU) according to the standard ISO 7027:2016. For every selected soil mass, the experiment was repeated thrice and an arithmetic mean of the three values considered. Variation of the soil mass with the resulting turbidity was obtained through a scatter plot followed by fitting of a linear regression line (Fig. 1). To compute the Kaolin clay masses (*C*) in mg/l required to obtain a resulting turbidity (*T*) in NTU, an equation of the fitted line or T=81.754×C was used. Coefficient of determination (*R*^2^) value of 0.9915 was obtained from the regression analysis. Since the *R*^2^ was close to one (1), it showed that the linear model adequately described the variation of *T* with *C*. The turbidity values were chosen between zero and 125 NTU. This range covered low, moderate and high turbidity values typical of surface water.

#### 2.3.2 Preparation of *M*. *indica* coagulation solution

To prepare coagulating solution, 5 g of each *M. indica* genotype powder was weighed using a weighing scale and dissolved in 100 mL of distilled water. This meant that 1 mL of distilled water contained 0.05 g of *M. indica* powder. The mixture was homogenized using a magnetic stirrer at a speed of 500 rpm for 30 mins in order to agitate the active coagulating protein component in each genotype. The resultant solution was filtered using a filter paper (porosity <8 μm) and funnel in a conical flask in order to obtain the coagulating solution. The resultant filtrate was used as the stock solution from which various volumes were used to treat the synthetic turbid water of a constant volume. The experiment was conducted at a room temperature.

#### 2.3.3 Performance of *M. indica* in turbid water coagulation

To investigate the turbidity removal efficacy, six (6) different beakers were each filled with 200 mL of the turbid water of a chosen turbidity (15, 65, or 120 NTU). To the turbid water, the coagulating (or stock) solution of a particular *M. indica* genotype (as described in section 2.3.2 was used) was added to the different beakers in varying volumes. One at a time, the volume of coagulating (or stock) solution poured onto the turbid water in each beaker included 5, 10, 15, 20, 25, and 30 mL. The resulting mixture in each beaker was homogenized using a mixer for 30 mins and left to coagulate for 12 hours. The supernatant solution was then poured in a sample bottle. The turbidity and pH of the supernatant solution were measured. The turbidity removal efficacy was obtained as the ratio of the difference between final and initial turbidity as a percentage of the initial turbidity. The above procedure was repeated thrice for each Mango variety and selected volume of coagulating solution and the average of the three values was considered for obtaining the turbidity removal efficacy.

## 3 Results and discussion

### 3.1 Protein contents

**Fig. 2:**
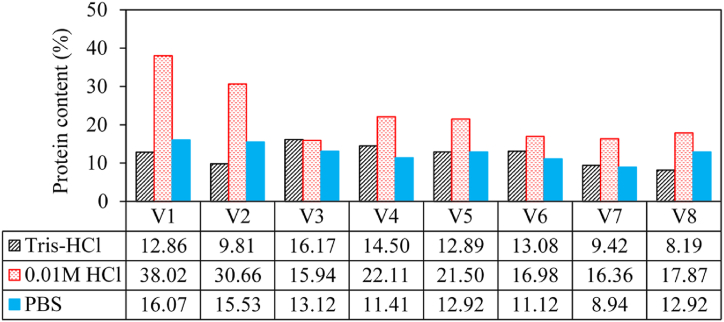
Protein content for the Mango varieties.

Fig. 2 shows results of protein contents from the various Mango varieties. Genotype V1 yielded the highest protein contents of 38.02 % and 16.07 % based on 0.01 M HCl and PBS extraction solvents, respectively (Fig. 2). However, V3 yielded the highest protein content (16.17 %) when Tris-HCl was used as the extraction solvent. Protein contents based on Tris-HCl and PBS were comparable and fell in the range 7–17 %. The coagulating active components believed to be proteins was found to constitute up to 58 % of the mango seed [13]. Using sodium hydroxide (NaOH) and NaCl extraction solvents, the coagulating active component of the mango seed kernel was found to be 25.1 % and 49.75 %, respectively [14]. Results from this study showed that the protein content of a plant part (such as the mango seed in this case) depends on the extraction solvent used.

In this study, the average pH values of Tris-HCL, PBS, 0.01 M HCl extraction solvents were 7.7, 7.9, and 2.9, respectively. The mean and standard deviation (Stdev) of the pH values of resulting mixtures of extraction solvents and *M*. *indica* solutions are summarized in Table 2. After adding the coagulant to Tris-HCl, the pH dropped from 7.7 (alkaline condition) to as low as 6.1 (acidic) using V1. The initial pH 2.9 (acidic condition) of 0.01 M HCl increased to a range varying from 5.7 (V7) to 6.2 (V2) after addition of the coagulant. This showed the tendency of the pH to shift from acidic towards neutral condition. When the coagulant was added to PBS, the pH of the resulting mixture ranged from 6.2 (V7) to 6.7 (V2) instead of the initial 7.9. These results show that *M*. *indica* seeds possess the ability to modify pH of the water being treated. The effect of pH on the extraction of protein from a plant was confirmed in several past studies [[15], [16], [17], [18]]. For instance, Oak leaves were demonstrated to show very low and high solubility at acidic (pH 2) and alkaline condition (pH 12), respectively, leading to maximum protein extraction of about 4 mg/g at pH 12 [15]. Though not considered in this study, temperature is another factor which can affect the extraction of protein from a plant [15,16]. Increasing temperature leads to decrease in protein concentration [17,18]. Very high temperature leads to breaking of hydrogen bonds thereby making the proteins to become unstable [15]. Maceration time, though not considered in this study, also influences the protein extraction. Increasing maceration time leads to a decrease in the amount extracted protein [15]. The need to comprehensively investigate the effects of the above various factors such as pH, temperature, and others while considering *M*. *indica* as a coagulant remains a proposition for a future research.

**Table 2:** Mean and standard deviation of the mixture containing protein extraction solvents and Mango variety sample.

	*M*. *indica* genotypes							
Metric	V1	V2	V3	V4	V5	V6	V7	V8
Solution of Tris-HCL and Mango variety sample								
Mean	6.1	6.8	6.7	6.7	6.7	6.4	6.2	6.3
Stdev	0.269	0.025	0.005	0.015	0.008	0.010	0.005	0.032
Solution of 0.01 M HCL and Mango variety sample								
Mean	5.8	6.2	6.1	6.1	6.1	6.1	5.7	6.0
Stdev	0.032	0.016	0.010	0.008	0.002	0.007	0.020	0.025
Solution of PBS and Mango variety sample								
Mean	6.4	6.7	6.5	6.5	6.6	6.4	6.2	6.3
Stdev	0.006	0.007	0.009	0.008	0.013	0.008	0.006	0.014

### 3.2 Turbidity removal efficacy

**Fig. 3:**
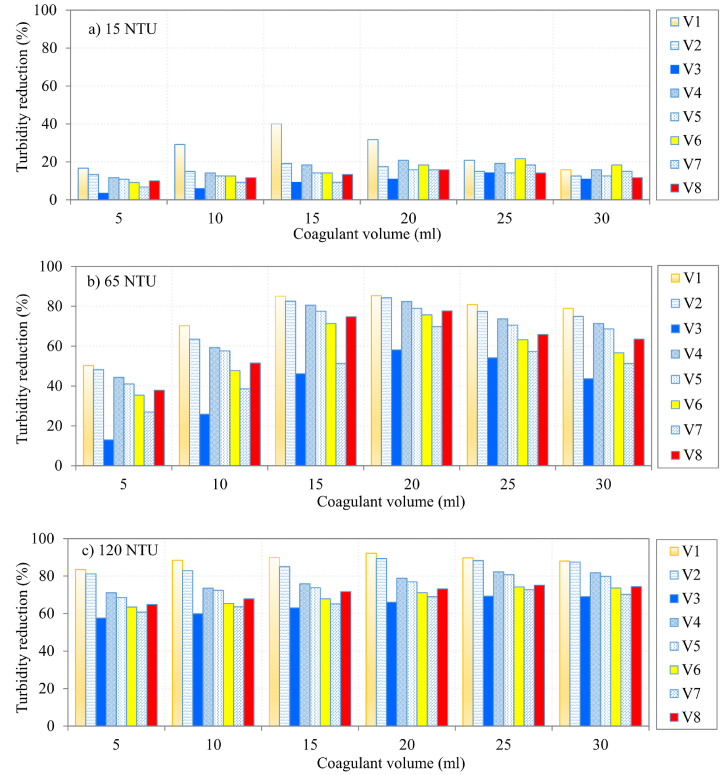
Efficiency of coagulation when initial turbidity was a) 15 NTU, b) 65 NTU, and c) 120 NTU.

Fig. 3 shows coagulation efficacy of the considered *M*. *indica* genotypes. Coagulation efficacy was generally found to increase with increasing turbidity. For instance, the efficacies of V1-based coagulant were 16.7, 50.3, and 83.5 % for 15, 65 and 120 NTU turbidity levels, respectively (Fig. 3a–c). The corresponding removal efficacies for V1 when the coagulant's volume was 20 mL were 31.7, 84.2, and 89.3 % for 15, 65 and 120 NTU turbidity levels, respectively.

For turbidity of 15 NTU, the coagulation efficacies of the considered *M. indica* genotypes (except V1) generally varied from 3.3 % to about 20 % (Fig. 3a). For turbidity of 65 NTU (Fig. 3b), the coagulation efficacies of a selected Mango variety increased with increasing coagulant volume from 5 to 20 mL. Thereafter, the turbidity removal efficacy reduced with further increase in coagulant volume. This similar observation was realized only for V1 and V2 when turbidity of 120 NTU was considered (Fig. 3c). This suggested that the optimum coagulant volume to treat 200 mL of 65 NTU turbidity was 20 mL. Given that for the experiment of this study, 1 mL of distilled water contained 0.05 g of *M. indica* powder, the optimum coagulant mass in the 20 mL was 1 g. On the other hand, the coagulation efficiencies of the other remaining varieties including V3, V4, V6, V7, and V8 increased as the coagulant's volume was incrementally varied from 5 to 30 mL. This showed that the optimal dosage for turbidity removal depends on the selected *M. indica* genotype as posited in this study.

For 120 NTU turbidity, the turbidity removal efficacies for the Mango varieties used in this study considering coagulant's volume over the range 5–30 mL varied from 57.5 % (for 5 mL of V3) to 92.2 % (20 mL of V1) (Fig. 3c). For 65 NTU turbidity, the corresponding percentages were 12.9 % (for 5 mL of V3) to 85.3 % (20 mL of V1) (Fig. 3b). Again for 15 NTU turbidity, the coagulation efficacies ranged from 3.3 % (for 5 mL of V3) to 40 % (20 mL of V1) (Fig. 3a). Nevertheless, the difference in coagulation efficacy of each *M. indica* variety between 65 NTU and 15 NTU turbidity levels was larger than that for 65 and 120 NTU. In summary, the largest coagulation efficacy was generally achieved using V1 considering both selected coagulant's volume and turbidity level (Fig. 3a–c) thereby indicating that V1 is the best performing variety as a coagulant. The performance of V1 can be linked to its high protein content as shown by two of the three protein extraction solvents (Fig. 2).

A number of past studies [10,13] investigated the efficacy of mango seed kernels for treating turbid water. In treating synthetic turbid water with 150 NTU turbidity, removal efficacy of up to 92 % was achieved under the optimum condition of 25 mL/L of mango at pH of 7.0 [10]. Furthermore, turbidity removal efficacy of 98.6 % was found under an optimum condition of 0.5 mL/L of mango seeds at pH of 13 to treat synthetic turbid water with turbidity values in the range 17.5–90 NTU [13]. The optimum turbidity removal of Mango seed was also achieved using dosage of 100 mg/L under pH of 6 or 9 [14]. The potential of *M*. *indica* with the 9 mL of the coagulant (under pH varying from 7.3 to 8.05) exhibited in removing water turbidity went up to 85.45 % [19]. Coagulation efficacy of Mango in turbidity removal has been shown to be comparable to that of Aluminium salts [19,20]. Furthermore, Mango performs better than other plants in turbidity removal [21].

As already seen from Table 2, the use of *M*. *indica* seeds can either reduce or increase pH. This depends on the chemical nature of the substance contributing to the turbidity of the polluted water. For instance, in this study, Kaolin clay was used to create turbid water. The pH of the clay soils used in this study were around 6.0. This was consistent with results from a previous study [22]. The pH values of Kaolin clay soils at Kajansi, Seeta, Nakawa, and Kawuku (as areas that are a few kilometers away from where the clay used for this study was obtained) were found to be 5.96 ± 0.034, 6.1 ± 0.034, 5.75 ± 0.034, and 6.4 ± 0.034, respectively [22]. These soils in the central region of Uganda especially around Kampala City are slightly acidic probably because of the natural acidity of the hydroxyl groups on silica sites [22,23]. The pH values of the treated water samples ranged from 6.7 (V1) to 7.2 (V7). The pH values were within the World Health Organization [24] recommended range of 6.5–8.5. It means that the nature of the substance contributing to the turbidity of the water should be understood as the pollutant can alter the pH of the treated water to become acidic or alkaline.

This study considered liquid form of the coagulant. However, powder form of the *M*. *indica* coagulant could also be used to treat turbidity. Past studies [9,25,26] showed that natural coagulants yield higher efficacies in the liquid than powder form. For instance, *Aloe vera* powder and liquid forms as organic coagulants were shown to yield turbidity removal efficiencies of 28.2 and 87.8 %, respectively [26]. Acorn in its powder and liquid form removed 71.6 and 84.8 %, respectively, of initial turbidity 13 NTU [9]. Both *Moringa Oleifera* and *Cactus Opuntia* performed better in their liquid than powder form when applied to treat turbid water [25].

## 4 Conclusions

Using 0.01 M HCl as the extraction solvent, the protein contents of the selected *M*. *indica* genotypes including Apple mango, Kate, Kent, Bire, Doodo red, Takataka, Kagoogwa, Tommy Atkins were 38.02 %, 30.66 %, 15.94 %, 22.11 %, 21.50 %, 16.98 %, 16.36 %, and 17.87 %, respectively. Protein contents of these *M. indica* genotypes based on Tris-HCl and PBS were lower than those obtained using 0.01 HCl and fell in the range 7–17 %. Thus, the choice of a particular method for extraction of coagulating active components influences the quantification of the protein content of a plant. Other factors which can influence protein extraction from *M. indica* include pH, temperature, and maceration time.

Apple mango was the best performing genotype as a coagulant due to its highest protein content compared to other genotypes. Coagulation efficacy of each *M. indica* genotype was generally found to increase with increasing turbidity or coagulant's concentration. For instance, the efficacies of 5 mL of *M. indica* coagulant solution made from 5 g of Apple Mango dissolved in 100 mL of distilled water were 16.7 %, 50.3 %, and 83.5 % for turbidity of 15, 65 and 120 NTU, respectively. The percentages of the initial turbidity 120 NTU removed using 5, 20, and 30 mL of Kent coagulant stock solution were 57.5, 66, and 69 %, respectively.

We believe that any other plant (apart from Mango) known to be a good coagulant will have differences (among the various genotypes) in the percentage of the coagulating active components understood to be proteins. Thus, further research is required as conducted in this study while considering other plants that could be used as natural coagulants.

By intensifying studies in line with the advancement for clean technology to replace chemical coagulants using natural solutions, we strongly recommend that some breakthrough research is required on the feasibility of scaling laboratory-based coagulation experiments into actual pilot water treatment system which can make use of plant extracts for turbidity removal.

## Funding

This research received no external funding.

## Data availability

Data used in this study will be made available on formal request to the corresponding author.

## CRediT authorship contribution statement

**Charles Onyutha:** Conceptualization, Data curation, Formal analysis, Investigation, Methodology, Project administration, Supervision, Visualization, Writing – original draft, Writing – review & editing. **Nancy Auma:** Conceptualization, Data curation, Formal analysis, Investigation, Methodology, Writing – original draft, Writing – review & editing.

## Declaration of competing interest

The authors declare that they have no known competing financial interests or personal relationships that could have appeared to influence the work reported in this paper.
